# Comment on ‘Anthropometric measurements and survival after prostate cancer diagnosis’

**DOI:** 10.1038/s41416-018-0161-3

**Published:** 2018-07-31

**Authors:** Luigino Dal Maso, Antonella Zucchetto, Carmen Stocco, Diego Serraino, Jerry Polesel

**Affiliations:** 1 Unit of Cancer Epidemiology, CRO Aviano National Cancer Institute, via Franco Gallini 2, 33081 Aviano, PN Italy; 2 Venetian Cancer Registry, Veneto Region, Passaggio Gaudenzio 1, 35131 Padua, Italy

**Keywords:** Cancer epidemiology, Risk factors

We read with great interest the recent paper by Farris et al.^1^ on the lack of significant associations between anthropometric measurements and prostate cancer prognosis. In particular, body mass index (BMI), as a measure of obesity, was neither associated with overall nor with prostate cancer-specific mortality. However, the hazard ratio (HR) was not adjusted for tobacco smoking, given the lack of effect modification of smoking, evaluated by testing the interaction between smoking and BMI. Nonetheless, tobacco smoking might have exerted a residual confounding, given its association with both leanness^2^ and worse prostate cancer prognosis.^3^

In a previous paper, we have already reported null associations between prostate cancer survival and BMI^4^ found in a cohort study with a similar design of that by Farris and colleagues.^1^ In order to provide further insights on the potential confounding role of tobacco smoking, we have re-analysed the data from that cohort study, investigating the effect of BMI in separate strata of smoking habits. Men with prostate cancer (*n* = 778; median age: 66 years), originally enroled, between 1995 and 2002, in a case–control study as cases were followed up through population-based cancer registries to assess their vital status and the eventual underlying cause of death.^3^ During a median follow-up of 12.7 years, 267 deaths were observed, of which 82 were due to prostate cancer. Information on lifestyle factors and anthropometric measurements were collected at cancer diagnosis through a validated questionnaire. After verification of proportionality assumptions, HRs of death and related 95% confidence intervals (CI) were computed using the Cox proportional hazard model.

In our cohort of men with prostate cancer, there was no significant association between BMI and all-cause mortality (Table 1). However, a potential modification effect of smoking habits emerged (*p* value for interaction = 0.03). Indeed, among never smokers, there was a clear pattern of increased risk of death (*p* value for trend = 0.02) with a statistically significant higher risk among men with BMI ≥30 kg m^−2^ versus those with BMI <25 kg m^−2^ (HR = 2.76; 95% CI: 1.26–6.06). Conversely, no significant trend in risk emerged among men who were former or current smokers. Prostate cancer-specific mortality showed a similar pattern with a higher risk of death for BMI ≥30 kg m^−2^ only among never smokers (HR = 4.24, 95% CI: 0.81–22.3) (Table 1). However, our study was under-powered to evaluate cause-specific mortality and this result was not statistically significant due to a very low number of observed events (*n* = 4).

**Table 1:** Anthropometric measurements at diagnosis in 778 men with prostate cancer in relation to all-cause mortality and prostate cancer-specific mortality, according to tobacco smoking habits at diagnosis

BMI (kg m^−2^)	Overall		Smoking habits					
			Never		Current		Former	
	Deaths/cases	HR (95% CI)^a^	Deaths/cases	HR (95% CI)^a^	Deaths/cases	HR (95% CI)^a^	Deaths/cases	HR (95% CI)^a^
All-cause mortality								
<25	96/261	1^b^	16/80	1^b^	29/65	1^b^	51/116	1^b^
25–<30	129/408	0.78 (0.59–1.01)	33/116	1.44 (0.78–2.68)	30/81	0.72 (0.42–1.24)	66/211	0.64 (0.44–0.93)
≥30	42/109	0.95 (0.65–1.38)	13/30	2.76 (1.26–6.06)	9/19	0.98 (0.45–2.13)	20/60	0.69 (0.40–1.18)
*χ*^2^_trend_		*p* = 0.41		*p* = 0.02		*p* = 0.58		*p* = 0.07
					*χ*^2^_interaction_: *p* = 0.03			
Prostate cancer-specific mortality								
<25	24/261	1^b^	3/80	1^b^	9/65	1^b^	12/116	1^b^
25–<30	44/408	0.99 (0.60–1.64)	9/116	1.47 (0.36–5.98)	11/81	0.84 (0.32–2.21)	24/211	0.84 (0.41–1.74)
≥30	14/109	1.12 (0.57–2.19)	4/30	4.24 (0.81–22.3)	5/19	1.65 (0.50–5.43)	5/60	0.65 (0.22–1.94)
*χ*^2^_trend_		*p* = 0.79		*p* = 0.11		*p* = 0.55		*p* = 0.44
					*χ*^2^_interaction_: *p* = 0.62			

^a^Estimated from Cox proportional hazard model, adjusted for age at diagnosis, year of diagnosis, region of residence, Gleason score, alcohol drinking habits, smoking habits, and smoking intensity, when appropriate.

^b^Reference category

A similar confounding effect of tobacco smoking was recently reported in the association between lifetime obesity and the risk of prostate cancer-specific mortality.^5^ Long-term weight gain (HR = 1.59 for >30 lb gained) and, suggestively, BMI >30 kg m^−2^ at diagnosis (HR = 1.23) were associated with lethal prostate cancer only among never smokers, whereas no association emerged in the whole cohort (HR = 1.16 and 1.09, respectively). Although death risk estimates were adjusted for smoking status, these findings suggest a potential residual confounding due to tobacco smoking.^5^ Furthermore, in their meta-analysis, Cao and Ma^6^ concluded that the reported J-shaped association between BMI and prostate cancer mortality was likely to be confounded by tobacco smoking. Therefore, the direct association between obesity and mortality we found in prostate cancer patients who were never smokers is likely to be real, and it is supported by biological plausibility. Indeed, beside possible delayed diagnosis in obese men, obesity may adversely impact the survival through metabolic, hormonal and inflammatory pathways.^5^

In our study, informations of anthropometric measurements were collected only at cancer diagnosis and no modifications of lifestyle factors were assessed thereafter. Nonetheless, changes in BMI were unlikely to have occurred following prostate cancer diagnosis, as well as modifications in smoking habits.^7^

These findings provide additional information to better understand the complex association between obesity and prostate cancer survival. Indeed, several correlated factors (including obesity, diabetes, physical activity and tobacco smoking) play concomitantly in the prognosis of prostate cancer patients, modifying one another’s association. Stratification according to potential confounders (e.g., smoking habits) should be always considered to explore the complex association between an outcome and the inter-correlated factors.^8^ In the light of our findings, Farris and colleagues could greatly contribute to the understanding of the real association between BMI and prostate cancer survival, presenting their results stratified by smoking habits, in particular for never smokers.

## Availability of data and material

Data are available for scientific purposes on request to the corresponding author
